# A case of hypogonadtropic hypogonadism due to hypophysitis discovered by secondary male infertility

**DOI:** 10.1002/iju5.12548

**Published:** 2022-10-22

**Authors:** Masatoshi Konishi, Yoko Maegawa, Masaru Tani, Toshihisa Asakura, Yujiro Hayashi, Yoichi Kakuta, Koichi Tsutahara, Kazuhiko Komori, Tetsuya Takao

**Affiliations:** ^1^ Department of Urology Osaka General Medical Center Osaka Japan; ^2^ Komori Clinic Osaka Japan

**Keywords:** hormone replacement therapy, hypogonadtropic hypogonadism, hypophysitis, secondary male infertility

## Abstract

**Introduction:**

The main causes of secondary male infertility are varicocele and aging. It is rarely caused by adult‐onset hypopituitarism. The onset of hypopituitarism is often due to brain tumors, trauma, surgery, or congenital disorders.

**Case presentation:**

A 29‐year‐old man was admitted to the hospital with complaints of decreased libido and semen volume, which lasted for 4 months. He had no abnormalities in adolescence and has a 2‐year‐old child. Blood tests showed low luteinizing hormone and follicle‐stimulating hormone, and semen tests showed azoospermia. Magnetic resonance imaging T1‐weighted images showed swelling and enhancement effect of the pituitary gland, and lymphocytic hypophysitis was suspected. After an Insulin‐thyroid‐stimulating hormone releasing hormone‐luteinizing hormone‐releasing hormone test, a decrease in luteinizing hormone/follicle‐stimulating hormone secretion was considered. We diagnosed hypogonadotropic hypogonadism due to lymphocytic hypophysitis. Currently, the patient is being treated with a hormone replacement therapy.

**Conclusion:**

We experienced a case of hypogonadotropic hypogonadism due to lymphocytic hypophysitis discovered by secondary infertility.

**Keynote message**

Adult‐onset hypopituitarism is rarely a cause of secondary male infertility. We presented a case of hypogonadotropic hypogonadism due to lymphocytic hypophysitis with the chief complaint of secondary male infertility.

**Abbreviations & Acronyms**
ACTH: adrenocorticotrophic hormone
FSH: follicle‐stimulating hormone
hCG: human chorionic gonadotropin
ICSI: intracytoplasmic sperm injection
IUI: intrauterine insemination
LH: luteinizing hormone
LHRH: luteinizing hormone‐releasing hormone
Micro‐TESE: microsurgical testicular sperm extraction
MRI: magnetic resonance imaging
TRH: thyroid‐stimulating hormone releasing hormone
TSH: thyroid‐stimulating hormone

## Introduction

The main causes of secondary male infertility are varicocele, and decreased semen volume, sperm motility, increased abnormal rate of sperm morphology and decreased sexual intercourse opportunities due to aging.^1^, ^2^ Adult‐onset hypopituitarism may cause secondary male infertility. However, the reported cases are rare. Hypogonadotropic hypogonadism is often due to hyperprolactinemia, pituitary lesions (tumor, granuloma, abscess), Cushing syndrome, pituitary irradiation, trauma or surgery, Kallmann syndrome, or Prader–Willi syndrome.^3^ Here, we report a case of hypogonadotropic hypogonadism due to lymphocytic hypophysitis diagnosed with secondary male infertility as the main complaint.

## Case presentation

**Fig. 1:**
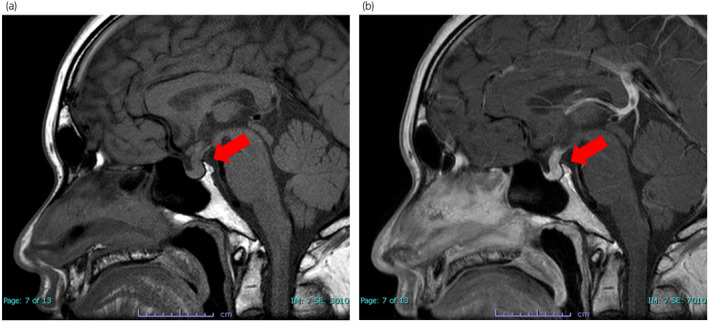
Radiological findings. (a) MRI T1‐weighted images reveals enlargement of the pituitary stem and no pituitary tumors. (b) MRI T1‐weighted images reveals uniformly enhanced pituitary stem, and lymphocytic hypophysitis is suspected.

A 29‐year‐old man complained of secondary male infertility. A semen test showed signs of azoospermia and he was referred to our department. His height was 163 cm, weight was 61.6 kg, and body mass index was 23.2 kg/m^2^. His libido and erectile capacity were declining. He was able to ejaculate, but his semen volume was decreasing. His penis and pubic hair were normal (G5 and PH5 at Tanner stage). The testes were 10 mL on both sides, varicocele was not evident. Blood test revealed a LH count of 0.7 mU/mL, a FSH count of 1.5 mU/mL, and a testosterone count of 1.4 ng/mL. We considered he was a possible case of adult‐onset hypogonadotropic hypogonadism because he had no problems with secondary sexual characteristics and had a first child. In addition, since there was no history of surgery or trauma, he was suspected to have interbrain or pituitary disease. After the Insulin‐TRH‐LHRH test, LH and FSH showed normal level, but the peak value exceeded the lower limit of normal, and it was suspected that there was a decrease in LH and FSH secretion ability from the pituitary gland. MRI T1‐weighted images showed pituitary stalk enlargement (Fig. 1a). After contrast enhancement, it was shown to be uniformly enhanced, and lymphocytic hypophysitis was suspected (Fig. 1b).

Based on these clinical findings, he was diagnosed with hypogonadotropic hypogonadism due to lymphocytic hypophysitis. He started self‐injecting 5000 units of hCG preparation twice a week. Thirteen months after the treatment, a semen test showed signs of azoospermia and rFSH preparation was added. Seventeen months after the first treatment, his subjective symptoms such as decreased libido have improved. No treatment was given for lymphocytic hypophysitis.

## Discussion

Secondary infertility is a situation in which parents fail to have more children after 1 year of trying. About one–third of secondary infertility cases originate in the male partner.^1^ Varicocele is the most common cause of secondary male infertility.^2^ Other causes include aging, impaired passage through the sperm, endocrine disorders, and hereditary disorders. Endocrine disorders may cause secondary male infertility. However, the reported cases are rare. Among endocrine disorders, hypogonadtropic hypogonadism is a form of hypogonadism that is due to a problem with the pituitary gland or hypothalamus. The causes of hypogonadotropic hypogonadism are classified as congenital and acquired.^3^, ^4^ Congenital causes include Kallman syndrome, Laurence–Moon–Biedl syndrome, Prader–Willi syndrome, gonadotropin deficiency, etc. Acquired causes include pituitary adenoma, hypophysitis, brain tumor surgery, radiation therapy, and brain trauma. Gomez *et al*. reported that rapid weight loss after sleeve gastrectomy was associated with hypogonadotropic hypogonadism.^5^ No rapid weight loss was observed in this patient.

We considered his case to be an acquired type because he had no problems with secondary sexual characteristics and had a first child. His testes were small at the first visit. Since he has a first child, we assume that his testes shrunk after hypogonadotropic hypogonadism. Based on the MRI, lymphocytic hypophysitis was thought to be the cause.

Lymphocytic hypophysitis is a condition in which the pituitary gland becomes infiltrated by lymphocytes, resulting in pituitary enlargement and impaired function. The frequency is 1 in 9 million, about 1% of pituitary diseases.^6^ The affected area is roughly classified into three types: anterior pituitary inflammation, posterior pituitary inflammation, and pan‐hypophysitis. Hormone tests, head contrast MRI, and pituitary biopsy are the main tests, but a biopsy is often not performed due to the invasive aspects. Treatment involves supplementation of reduced hormones, and if pituitary enlargement causes visual impairment, steroid therapy or transsphenoidal sinus pituitary mass resection is performed.^6^, ^7^, ^8^

The cause of lymphocytic hypophysitis is not clear, but an association with the autoimmune mechanism is speculated. Chalan *et al*. reported its association with Th17 cells.^9^ It has been suggested that IL17 produced from Th17 may induce various inflammatory mediators, provoke an inflammatory response, and play a central role in the pathophysiology of hypophysitis.

The first treatment of infertility due to hypogonadotropic hypogonadism is hormone replacement therapy. Rastrelli *et al*. reported that the sperm appearance rate was 47% with the hCG preparation alone, 80% with the hCG and FSH preparation.^10^ It is reported that in cases of oligospermia after hormone replacement therapy, children are conceived with IUI or ICSI. Even in cases of azoospermia after hormone replacement therapy, it is reported that sperm that are obtained by micro‐TESE and ICSI leads to pregnancy.^11^, ^12^

In this case, the patient had started hormone therapy with the hCG and was still azoospermic upon semen examination after the treatment. He had just added the rFSH preparation and we will need to continue to monitor his progress.

## Conclusion

This is the first report of hypogonadtropin hypogonadism due to hypophysitis discovered by secondary male infertility known to us. It is necessary to continue to accumulate data on the treatment results for secondary male infertility.

## Author contributions

Masatoshi Konishi: Conceptualization; data curation; project administration; writing – original draft. Tetsuya Takao: Conceptualization; project administration; supervision; writing – original draft. Yoichi Kakuta: Conceptualization; supervision. Yujiro Hayashi: Conceptualization; supervision. Masaru Tani: Conceptualization; supervision. Toshihisa Asakura: Conceptualization; supervision. Yoko Maegawa: Conceptualization; supervision. Kazuhiko Komori: Resources; supervision. Koichi Tsutahara: Conceptualization; supervision.

## Conflict of interest

The authors declare no conflict of interest.

## Approval of the research protocol by an Institutional Reviewer Board

Not applicable.

## Informed consent

Informed consent was obtained from patient.

## Registry and the registration no. of the study/trial

Not applicable.
